# Do patients get better? A review of outcomes from a crisis house and home treatment team partnership

**DOI:** 10.1192/bjb.2018.105

**Published:** 2019-06

**Authors:** Mohsin Faysal Butt, David Walls, Rahul Bhattacharya

**Affiliations:** 1 Centre for Psychiatry, Barts and The London School of Medicine and Dentistry, Queen Mary University of London, UK; 2 Look Ahead Housing and Care, UK; 3 East London NHS Foundation Trust, UK

**Keywords:** Crisis house, outcome PROM CROM, Health of the Nation Outcome Scales, DIALOG, home treatment team

## Abstract

**Aims and method:**

The Tower Hamlets Crisis House (voluntary sector), in partnership with the local home treatment team, offers a brief residential alternative to psychiatric hospital admission. Here, we review clinician-reported (Health of the Nation Outcome Scales; HoNOS) and patient-reported (DIALOG) outcome scores collected from successive admissions between June 2015 and December 2016, to assess the effectiveness of the service model. We identified 153 successive admissions, and of these, 85 (55.6%) and 91 (59.5%) patients completed both admission and discharge DIALOG and HoNOS questionnaires, respectively. We analysed ten out of twelve HoNOS domains and eight patient-reported outcome measure DIALOG domains.

**Results:**

We found a statistically significant improvement in nine out of ten domains of HoNOS and three out of eight domains of DIALOG.

**Clinical implications:**

A partnership between a home treatment team and crisis house can result in positive outcomes for patients, as determined by both clinicians and patients.

**Declaration of interest:**

None.

## What is a crisis house?

Over the past decade, a nationwide drive to reduce and consolidate in-patient psychiatric beds across the UK National Health Service (NHS) has resulted in a concomitant growth in alternative models of intensive care in community settings, including an increase in the number of crisis houses.^1^ A crisis house offers provision for people who find themselves in a mental health crisis as a community-based alternative to hospital admission.^2^ The Tower Hamlets Crisis House (THCH) offer a short-term, community-based service for people who find themselves in significant mental distress, which is recognised to be potentially less stigmatising, coercive and institutionalised.^3^ A mental health crisis may be because of a variety of reasons, ranging from suicidal behaviour with or without intent, panic attacks or extreme anxiety, psychotic episodes or other behaviours that can potentially endanger the patients themselves or others.^4^

## Outcomes in Mental Health

Healthcare outcomes are the results of care in terms of patients' health over time.^5^ Outcomes can be assessed by the patient, in the form of a patient-reported outcome measure (PROM), or by the clinician, in the form of a clinician-reported outcome measure (CROM). Over the past decade, increasing emphasis has been placed on medical interventions that yield high value for patients, with value defined as the health outcomes achieved per unit currency spent.^5^ If value improves, both patients and commissioners can benefit as the economic sustainability of the healthcare system increases. Although home treatment team (HTT) services are known to have inexpensive resource utilisation and foster better patient experiences,^6^ there is little research exploring their outcomes, particularly PROMs. The DIALOG intervention is an 11-item PROM that assesses patients' well-being on a 7-point Likert scale. Only the first eight questions of the DIALOG intervention assess patient-reported outcomes, whereas the last three domains assess patient experiences. The Health of the Nation Outcome Scales (HoNOS) intervention is a 12-item CROM intervention assessing four domains on a five-point Likert scale (0–4): behavioural problems, impairment, symptomatic problems and social problems.

## Aims

To our knowledge, there is a paucity of agreed methodology for the analysis of CROM data.^7^^,^^8^ Similar challenges exist in analysing PROM data, given no nationally agreed methodology exists to assess these outcomes. The aim of this study was to measure the effectiveness of the THCH, using routine collection of PROM and CROM data.

## Method

Data were collected as part of a routine service key performance indicator by the voluntary sector provider in partnership with the NHS trust and reported to the commissioners. The study received ethical approval as a service evaluation from the East London NHS Foundation Trust because the process did not involve any additional data collection or patient contact.

In this retrospective case series, we reviewed HoNOS and DIALOG data from successive patients admitted to the THCH at both admission and discharge (two-point data), between June 2015 and December 2016. Data were collected from quarterly reports submitted as a part of quarterly reporting. To avoid double-counting, e.g. in patient episodes that spread over more than one quarter, data were reconciled to ensure patients who were admitted and discharged over two different quarters were identified as single patients and double-counting was therefore avoided.

For the analysis, the authors compared HoNOS (CROM) and DIALOG (PROM) ratings at admission and discharge. Therefore, we did not analyse the three patient-reported experience measures in the DIALOG intervention (questions 9–11). This study did not assess domain four (cognitive problems) of the HoNOS, as most of the patients presenting to the THCH did not have cognitive difficulties. We also decided not to assess domain eight of the HoNOS intervention (other mental and behavioural problems: specify A, B, C, D, E, F, G, H, I or J, where A is phobic, B is anxiety, C is obsessive–compulsive, D is mental strain/tension, E is dissociative, F is somatoform, G is eating, H is sleep, I is sexual and J is other, specify) because of the heterogeneity of conditions that are assessed in this domain. As a secondary outcome measure, the number of patients who required acute hospital admission or terminated their treatment in an unscheduled manner over this period were noted.

Results are displayed as the means ± s.e.m. Statistical significance between the two-point PROM and CROM data-sets were determined by a paired *t*-test. All statistical tests were performed with GraphPad Prism, version 6.0d for MAC OS (GraphPad Software Inc., San Diego, CA, USA). Statistical significance was assumed when differences were at *P* < 0.05.

## Results

**Fig. 1:**
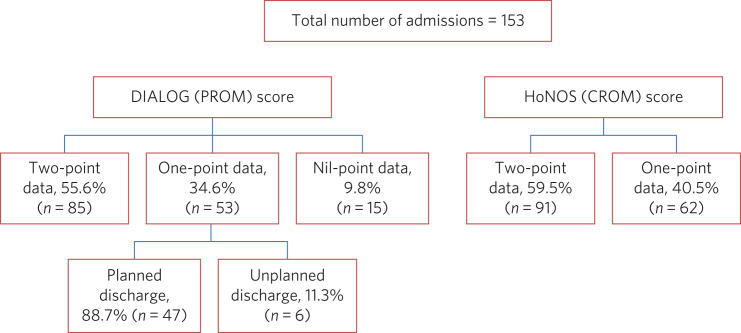
A flow diagram of patients in the study. CROM, clinician-reported outcome measures; DIALOG, PROM, patient-reported outcome measures; HoNOS, Health of the Nation Outcome Scales.

**Fig. 2:**
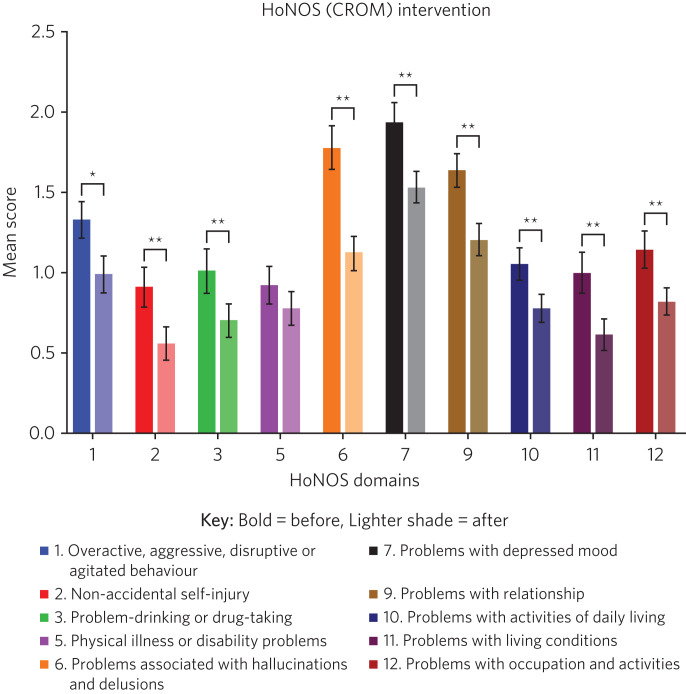
Outcomes of the HoNOS (CROM) score (*n* = 91). A lower mean score indicates a better psychiatric profile. The mean has been plotted along with ±s.e.m. CROM, clinician-reported outcome measures; HoNOS, Health of the Nation Outcome Scales. *P < 0.05, **P < 0.005.

**Fig. 3:**
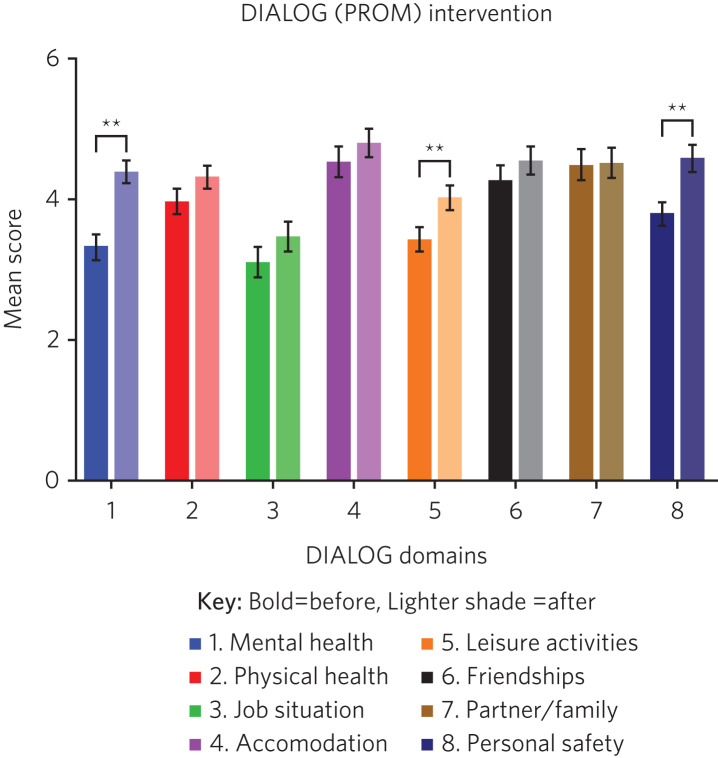
Outcomes of the DIALOG (PROM) score (*n* = 85). A higher mean score indicates a better psychiatric profile. The mean has been plotted along with ±s.e.m. DIALOG, PROM, patient-reported outcome measures. **P < 0.005.

There were 153 admissions during the time period. Two-point DIALOG and HoNOS data were available for 85 (55.6%) and 91 (59.5%) admissions, respectively. Among those for whom two-point DIALOG data were unavailable (*n* = 68): 47 customers did not complete the discharge PROM but had a planned discharge, 6 customers did not complete the discharge PROM as they were either admitted to hospital or abandoned occupancy, and for 15 patients there was no admission or discharge DIALOG score (see Fig. 1). Two-point and one-point HoNOS data were available for 91 (59.5%) and 62 patients (40.5%), respectively. There was >85% matching in the cohort of patients who completed the two-point DIALOG and HoNOS interventions. For HoNOS scores, we show a statistically significant improvement in nine out of ten assessed domains, where domain five (physical illness or disability problems) was the only outcome that did not reach statistical significance (see Fig. 2). Among the eight DIALOG scores assessed in this analysis, we show a statistically significant improvement in domains one (mental health), five (leisure activities) and eight (personal safety) (see Fig. 3). Comparing similar domains in the HoNOS and DIALOG (see Table 1), we show some degree of mirroring in the clinician and patient responses, although there is some discrepancy, e.g. comparison numbers four, seven, nine and ten.

**Table 1:** A comparison of similar domains in the HoNOS and DIALOG interventions

Comparison number	Domains assessed in the HoNOS intervention^a^	HoNOS *P* value	Domains assessed in the DIALOG intervention^b^	DIALOG *P* value	Relationship between HoNOS and DIALOG scores
1	Domain one: Overactive, aggressive, disrupted or agitated behaviour	*	Domain eight: How satisfied are you with your personal safety? Domain one: How satisfied are you with your mental health?	** **	Yes
2	Domain two: Non-accidental self-injury	**	Domain eight: How satisfied are you with your personal safety?	**	Yes
3	Domain three: Problem-drinking or drug-taking	**	Domain one: How satisfied are you with your mental health?	**	Yes
4	Domain five: Physical illness or disability problems		Domain two: How satisfied are you with your physical health?		No
5	Domain six: Problems with hallucinations and delusions	**	Domain one: How satisfied are you with your mental health?	**	Yes
6	Domain seven: Problems with depressed mood	**	Domain one: How satisfied are you with your mental health?	**	Yes
7	Domain nine: Problems with relationships	**	Domain six: How satisfied are you with your friendships? Domain seven: How satisfied are you with your partner/family?		No
8	Domain ten: Problems with activities of daily living	**	Domain five: How satisfied are you with your leisure activities?	**	Yes
9	Domain 11 Problems with living conditions	**	Domain four: How satisfied are you with your accommodation?		No
10	Domain 12 Problems with occupation and activities	**	Domain three: How satisfied are you with your job situation?		No

DIALOG, PROM, patient-reported outcome measures; HoNOS, Health of the Nation Outcome Scales.

a. This study did not assess domain four (cognitive problems) or domain eight (other mental and behavioural problems: specify A, B, C, D, E, F, G, H, I or J, where A is phobic, B is anxiety, C is obsessive–compulsive, D is mental strain/tension, E is dissociative, F is somatoform, G is eating, H is sleep, I is sexual and J is other, specify; all items are scored on a range from zero to four).

b. This study did not assess domain nine (How satisfied are you with your medication?), domain ten (How satisfied are you with the practical help you receive?) or domain 11 (How satisfied are you with the consultations with mental health professionals?). All domains are assessed on a range from one to seven.

* *P* < 0.05, **P < 0.005.

## Discussion

Over the past decade, commissioning in health has rightly turned its focus from commissioning for activity towards commissioning for outcomes. The Five Year Forward View for Mental Health stresses the importance of funding psychiatric services that have transparency around quality and outcomes, and suggested these should be in place by 2017–2018 for adult mental health services.^9^

In recent years, the patient–doctor relationship has evolved: from a paternalistic approach to one that is more collaborative with increased respect for patient autonomy.^10^ Given this evolution in the healthcare model, there is increasing recognition of the importance of involving patients in the development and evaluation of healthcare service delivery and quality improvement. PROMs are the tools that have been developed to ensure both a valid and reliable measurement of patient-reported outcomes. PROMs are directly reported by the patient without interpretation of the patient's response by a clinician or anyone else and pertain to the patient's functional status associated with healthcare or treatment.^11^ Capturing both PROM and CROM data in clinical practice provides a more complete understanding of the impact of a healthcare intervention.

HoNOS is mandated as the nationally recommended generic CROM, to be administered by mental health professionals.^9^ Although HoNOS has been around for 20 years,^12^ currently no universally agreed methodology for analysing this score exists in the literature.^8^^,^^13^ The DIALOG intervention was developed as part of a multicentre trial developed from Mensa, and then further developed as a solution-focused therapy tool.^14^ The DIALOG intervention is suggested by NHS England as an effective PROM intervention, but there are several alternatives, including the Questionnaire about the Process of Recovery and Short Warwick & Edinburgh Mental Well Being Scale.^9^ The analysis of PROM scores around DIALOG is in its infancy.

To our knowledge, this is the first report to systematically compare outcomes of the HoNOS and DIALOG interventions in a crisis house and HTT partnership. Given no standardised approach to analyse HoNOS and DIALOG data-sets exists in the literature, we analysed each item in the HoNOS and DIALOG interventions individually to achieve maximum granularity of data, as well as to allow us to compare similar fields in DIALOG and HoNOS, which would permit a degree of triangulation of clinician and patient perspectives. Our study shows that improvement in CROM scores is not always reflected by an improvement in PROM scores.

It is encouraging that mental health (DIALOG domain one) and personal safety (DIALOG domain eight) – the domains in which a mental health crisis service would be expected to have maximum impact – both feature statistically significant improvement in PROM scores. This finding is supported by statistically significant improvement in similar CROM scores assessing overactive, aggressive, disrupted or agitated behaviour (HoNOS domain one); overactive, aggressive, disrupted or agitated behaviour (HoNOS domain one); non-accidental self-injury (HoNOS domain two); problem-drinking or drug-taking (HoNOS domain three); problems with hallucinations and delusions (HoNOS domain six) and problems with depressed mood (HoNOS domain seven).

Broadly speaking, there are four models of community base community-based crisis services:^3^ (a) clinical crisis houses, providing residential services with staff onsite through the night and have a high level of clinical staff involved in providing onsite care; (b) specialist crisis houses, which share similar features to clinical crisis houses but are aimed at specific groups such as women and people with early psychosis; (c) crisis team beds, which provide a small number of beds aimed at short stays and are fully integrated with Crisis resolution and home treatment (CRHT) teams and (d) non-clinical alternatives, which are mainly managed by the voluntary sector with few clinical staff but many have also forged strong links with CRHT teams. Not all crisis houses have the same degree of collaboration with CRHTs, nor do they all offer residential support; for example, the Dial House in Leeds, UK.^15^ The THCH, established in 2010, is a partnership between the voluntary sector provider Look Ahead and East London NHS Foundation Trust (ELFT). The THCH service is embedded within the HTT, which ‘gate-keeps’ all admissions to the accommodation. There were initially five beds in the facility, which expanded to a ten-bed service in 2012. We would consider our model a hybrid of model (a) and (c) and our results indicate that such a model is effective in facilitating patient recovery. Given the heterogeneity of crisis house models, our outcomes cannot be generalised to other models of crisis house.

Our study is not without limitations, one being the limited sample size. Indeed, patients have a right to refuse to complete the DIALOG questionnaire, which explains the relatively little DIALOG data compared with HoNOS (85 *v.* 91). Although we attempted to administer the HoNOS scale to all patients, this could be done more predictably at admission: unscheduled discharges or very short admissions limited the opportunity to obtain discharge, and consequently, two-point data.

Unfortunately, the data used to analyse CROM and PROM outcomes did not include demographic details or ICD-10 codes. This information would have clarified the groups of individuals who most benefitted from the crisis house intervention. A previous multicentre study comparing the crisis house model to an in-patient psychiatric service noted that patient populations using both services were different with regards to gender, ethnicity and ICD 10.^16^ This dissimilarity could mean crisis houses are not a ‘true’ alternative to hospital admission, as the crisis house service could be treating patients who are not as unwell. This is an issue that does not affect the THCH: in a separate analysis performed by our group in 2015, we reviewed the patient profiles of crisis house clients (*n* = 299) and compared these with admissions to an acute in-patient ward (*n* = 677), and found that patients matched on gender, ethnicity and ICD-10 code (results were presented as a poster at the RCPsych International Congress^17^).

For (the majority of) patients who demonstrated an improvement in HoNOS and DIALOG scores, we are unable to comment on the precise intervention, or indeed the interplay of interventions, that facilitated improvement in their mental health. Was it being in a safe environment, the therapeutic relationship, pharmacotherapy, practical support or natural resolution of their social crises? In the absence of specific documentation of diagnoses, interventions offered and a ‘control’ group (e.g. an in-patient population), we are unable to discern the elements of the crisis house admission that were effective. It is also possible that this study has overestimated the impact of a crisis house intervention in the HoNOS and DIALOG scores, given patients for whom two-point data is available are more likely to have had a favourable course of treatment, as they would have been more engaged with staff and not made an unscheduled departure or required acute hospital admission.

In conclusion, we report evidence that a crisis house and HTT partnership can result in favourable results particularly around patients' mental health and safety as assessed by both the patient and clinician. Our findings support the effectiveness of a novel partnership model, supporting its continuance, and providing data to help mental health commissioners elsewhere in determining their local model of crisis care. Despite the study's limitations, its findings are worth disseminating, given that the evidence base for HTTs is inadequate and is even less established for crisis houses. Furthermore, the routine use of clinical outcome measurements in adult mental health remains patchy, despite significant national drivers. Our findings make a sizeable contribution to the limited literature describing the crisis house service, which is often poorly understood and infrequently commissioned. We hope this study encourages similar services to routinely collect and analyse PROM and CROM scores to develop a rich evidence base in this field.

## About the authors

**Mohsin Faysal Butt** is a medical student at Barts and The London School of Medicine and Dentistry, UK. **David Walls** is manager of Tower Hamlets Crisis House service, Lookahead Housing, UK. **Rahul Bhattacharya** is a consultant psychiatrist with the Tower Hamlets Home Treatment Team and clinical lead for Crisis House, East London NHS Foundation Trust, UK.
